# ROR2 homodimerization is sufficient to activate a neuronal Wnt/calcium signaling pathway

**DOI:** 10.1016/j.jbc.2023.105350

**Published:** 2023-10-12

**Authors:** Raul Riquelme, Laura Li, Abigail Gambrill, Andres Barria

**Affiliations:** 1 Department of Physiology and Biophysics, University of Washington School of Medicine, Seattle, Washington, USA; 2 Neuroscience Undergraduate Program, University of Washington, Seattle, Washington, USA

**Keywords:** Wnt signaling, Wnt pathways, ROR receptors, receptor tyrosine kinase, neurobiology, cell signaling, NMDA receptor, glutamate receptor, neurophysiology, neuroscience

## Abstract

Wnt signaling plays a key role in the mature CNS by regulating trafficking of NMDA-type glutamate receptors and intrinsic properties of neurons. The Wnt receptor ROR2 has been identified as a necessary component of the neuronal Wnt5a/Ca^2+^ signaling pathway that regulates synaptic and neuronal function. Since ROR2 is considered a pseudokinase, its mechanism for downstream signaling upon ligand binding has been controversial. It has been suggested that its role is to function as a coreceptor of a G-protein–coupled Wnt receptor of the Frizzled family. We show that chemically induced homodimerization of ROR2 is sufficient to recapitulate key signaling events downstream of receptor activation in neurons, including PKC and JNK kinases activation, elevation of somatic and dendritic Ca^2+^ levels, and increased trafficking of NMDARs to synapses. In addition, we show that homodimerization of ROR2 induces phosphorylation of the receptor on Tyr residues. Point mutations in the conserved but presumed nonfunctional ATP-binding site of the receptor prevent its phosphorylation, as well as downstream signaling. This suggests an active kinase domain. Our results indicate that ROR2 can signal independently of Frizzled receptors to regulate the trafficking of a key synaptic component. Additionally, they suggest that homodimerization can overcome structural conformations that render the tyrosine kinase inactive. A better understanding of ROR2 signaling is crucial for comprehending the regulation of synaptic and neuronal function in normal brain processes in mature animals.

Wnt signaling is a highly conserved signal transduction mechanism, which consists of multiple signaling cascades, each with distinct downstream effects and regulatory components. It plays crucial roles in regulating the embryonic development of metazoans (1, 2, 3), while dysregulation of this mechanism is also implicated in many human cancers (4, 5). Wnt proteins and their receptors are present in the mature central nervous system of mammals, where they participate in the regulation of axon pathfinding, axon remodeling, dendrite morphogenesis, and synapse formation (6, 7, 8, 9, 10). Several genes regulating Wnt signaling pathways have been associated with postdevelopmental onset neuropathologies, including schizophrenia (11, 12), bipolar disorder (13, 14), and Alzheimer’s disease, which has been proposed to involve deregulation of Wnt signaling as an etiological cause (15, 16, 17).

Our previous studies have identified a neuronal Wnt/Ca^2+^ signaling pathway that specifically upregulates synaptic currents mediated by NMDA-type glutamate receptors (NMDARs). This upregulation, in turn, facilitates synaptic plasticity, which is a cellular and molecular model of memory formation (18). The signaling cascade is triggered by Wnt5a, a Wnt associated with β-catenin–independent signaling. It is expressed in the mature hippocampus of rodents and is necessary for the proper dendritic arborization of principal neurons (6). Our research has identified ROR2, a tyrosine kinase–like receptor, as the necessary Wnt receptor that initiates the signaling cascade. Activation of the ROR2 receptor in neurons leads to an elevation in intracellular Ca^2+^, activation of PKC, and activation of c-Jun N-terminal kinase (JNK). This, in turn, results in an enhanced trafficking of GluN2B-containing NMDARs to the surface of the neuron and an increase in the number of NMDARs at synapses (19, 20).

ROR2 is a transmembrane receptor that plays a crucial role in the development and maintenance of various tissues in the body, including bones, connective tissue, and the nervous system. Additionally, it participates in several cellular processes, such as morphogenesis, cell proliferation, cell differentiation, cell migration, and cell death (21, 22, 23). The receptor has a single transmembrane domain with a cysteine-rich domain (CRD) in the extracellular N terminus and a tyrosine kinase–like domain in the intracellular C terminus. ROR2 is often considered a pseudokinase, despite biochemical evidence that it can phosphorylate itself or other substrates (24, 25, 26), and the fact that its tyrosine kinase domain (TKD) adopts a conformation similar to that of other kinases, albeit with unique stabilizing interactions and a few amino acid substitutions in key domains necessary for catalytic activity (27).

The Frizzled receptors (FZDs) are the most studied Wnt receptors. They belong to the superfamily of G protein–coupled receptors and can form complexes with other membrane proteins that act as coreceptors, including LRP5/6 as well as with ROR2 receptors (28, 29, 30). Several FZD are present in the hippocampus in juvenile rodents (31, 32, 33, 34) but whether they are also involved in the activation of the neuronal Wnt5a/Ca^2+^ pathway is not known.

In this study, we tested the hypothesis that homodimerization of ROR2 is sufficient to initiate the neuronal Wnt/Ca^2+^ signaling cascade that regulates the trafficking of NMDARs toward synapses. Biochemical evidence suggests that ROR2 can homodimerize in human cartilage tissue and human mesenchymal stem cells (25, 35). Our findings show that chemically induced homodimerization of ROR2 is sufficient to activate the Wnt/Ca^2+^ signaling cascade that regulates important neuronal functions. Furthermore, we examined the presumed tyrosine kinase activity of ROR2 and concluded that homodimerization of the receptor most likely activates an intrinsic kinase activity that phosphorylates the receptor and is necessary for downstream signaling.

## Results

This study explores the mechanisms of activation and signaling of ROR2 induced by Wnt5a. We tested the hypothesis that homodimerization of ROR2 induced by Wnt5a is sufficient to activate the neuronal Wnt/Ca^2+^ signaling pathway, which in turn leads to an increase in the trafficking of NMDARs toward synapses.

### ROR2 homodimerization induced by Wnt5a

Considering ROR2’s similarities with receptor tyrosine kinases (23, 26, 36), we first investigated whether Wnts can induce homodimerization of ROR2 receptors. To do this, we specifically utilized Wnt5a, a Wnt associated with β-catenin–independent signaling. Wnt5a initiates the neuronal signaling cascade, affecting synaptic function by increasing the trafficking of NMDA-type glutamate receptors (18, 19, 20).

**Figure 1:**
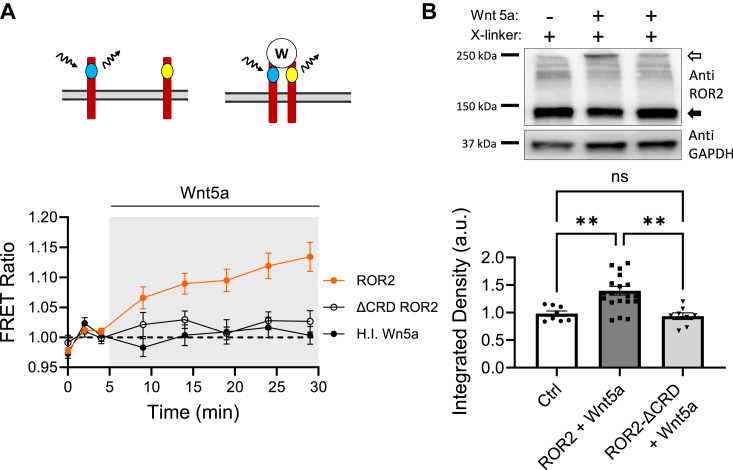
**ROR2 homodimerization.***A*, optical determination of ROR2 homodimerization. FRET ratio of YFP-ROR2 and CFP-ROR2 receptors cotransfected in HEK-293 cells and stimulated with 250 ng/ml Wnt5a or heat-inactivated Wnt5a. Z stack images were taken every 2 min during baseline. After stimulation, cells were sampled every 5 min. ROR2 (*red circles*; n = 105) and ROR2-ΔCRD (*open circles*; n = 46) were stimulated with Wnt5a. ROR2 was also stimulated with control Wnt5a heat inactivated (H.I. Wnt5a; n = 67). Here and through the paper, data is presented as mean ± s.e.m. *B*, biochemical determination of ROR2 homodimerization. *Top*, example of ROR2 immunoblot from HEK-293 cells expressing ROR2 (*left* and *middle lanes*) and ROR2-ΔCRD (*right lane*). Cells were stimulated for 15 min with 250 ng/ml Wnt5a and surface proteins crosslinked with BS3. The *open arrow* indicates crosslinked ROR2. *Black arrow* indicates ROR2 monomer. *Bottom* gel, GAPDH immunoblot of same samples. *Bottom*, quantification of *upper* band intensity normalized to crosslinked but nonstimulated cells (Ctrl n = 8) present in the same gel from cells expressing ROR2 and stimulated with Wnt5a (n = 20) or cells expressing ROR2-ΔCRD and stimulated with Wnt5a (n = 8). Statistical significance was determined using one-way ANOVA (F = 10.63; *p* < 0.001) and Tukey’s multiple comparisons test. *Asterisks*, ∗∗ *p* < 0.01. CRD, cysteine-rich domain.

To determine whether ROR2 homodimerizes upon stimulation with Wnt5a, we used two different assays. First, we transiently coexpressed optically tagged ROR2 receptors, CFP-ROR2 and YFP-ROR2, in HEK-293 cells and measured FRET before and after stimulation with Wnt5a (Fig. 1*A*, top). Although this approach cannot establish stoichiometry, it has the advantage of being applicable *in vivo* and is a useful way to test if Wnt5a can bring together at least two ROR2 molecules. Addition of recombinant Wnt5a to the bath increased FRET, indicating that optically tagged receptors were coming closer (Fig. 1*A*, bottom). Control heat-inactivated Wnt5a caused no changes in FRET. To test for a ligand-specific effect, we created a mutant of optically tagged ROR2 receptors that lacks the CRD, a common element present in FZD and ROR receptors, and necessary for ligand interaction (37, 38, 39). This mutant receptor (ROR2-ΔCRD) failed to multimerize when stimulated by Wnt5a, as indicated by the lack of FRET increase (Fig. 1*A*).

For the second assay, we performed a crosslinking assay for membrane proteins using bifunctional cross-linker BS3 with an arm length of 11.4 Å. We conducted this assay in two types of cells: HEK-293 cells expressing recombinant ROR2 for 24 h and dissociated hippocampal neurons to assess homodimerization of ROR2 receptors (Figs. 1*B* and 7*A*). We stimulated the cells with Wnt5a for 15 min before adding the crosslinking reagent to the media. The crosslinking reaction was stopped after 45 s, and the cells were homogenized. The proteins were then separated by SDS-PAGE, and the samples were immunoblotted using an ROR2-specific antibody (DSHB). As shown in Figure 1*B* (top panel), a certain amount of ROR2 is crosslinked in the absence of stimulation, as expected for overexpressed membrane proteins, and indicated by a shift in the molecular weight of ROR2 from ∼130 KD (lower band) to ∼250 KD (upper band), suggesting homodimerization of the receptor. We used the integrated density of this basal signal on each experimental repetition to normalize the values of other samples in the same gel. Stimulation with Wnt5a increased the proportion of crosslinked ROR2, indicating that two molecules of ROR2 came together. This shift was not observed when the ROR2 mutant lacking the binding site for Wnt5a (ROR2-ΔCRD) was expressed. Together, these two experimental approaches strongly suggest that Wnt5a induces homodimerization of ROR2.

### Tyrosine phosphorylation of ROR2 and downstream signaling

ROR2 is phosphorylated upon activation, leading to initiation of downstream signaling cascades. However, whether phosphorylation occurs at Ser/Thr or Tyr residues is dependent on the ligand and cellular context (24, 29, 40). This suggests that context-specific kinases could be responsible for some ROR2 phosphorylation. An analysis of the amino acid sequence of the tyrosine kinase domain of ROR receptors reveals that ROR2 and ROR1 have five and seven amino acid substitutions, respectively, in key motifs of the consensus sequence for tyrosine kinases (26). The identified amino acid substitutions are considered critical for the ROR receptors to exhibit any catalytic activity. In addition, structural studies show that the TKD of ROR2 has a conformation like that of inactive insulin tyrosine kinase (27). Thus, ROR2 has been classified as a pseudotyrosine kinase. However, biochemical evidence suggests that point mutations substituting a conserved residue, K507, typically involved in ATP binding in tyrosine kinases, can impede the phosphorylation of the receptor and downstream targets (24). In addition, *in vitro* autophosphorylation assays of truncated ROR2 and ROR1 showed that ROR2, but not ROR1, was able to exhibit kinase activity (26).

**Figure 2:**
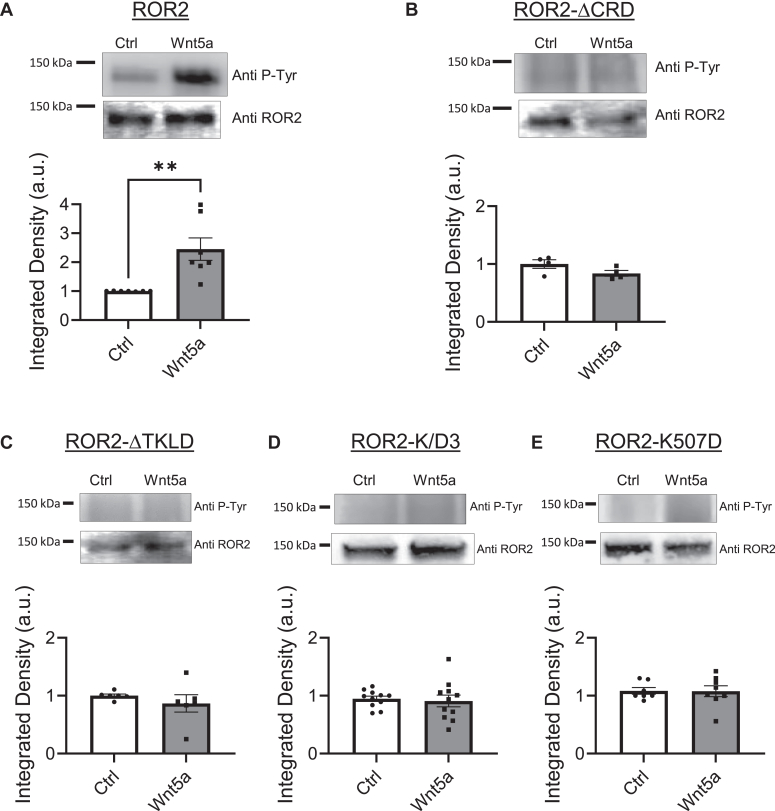
**Tyr phosphorylation of ROR2.***A*, Tyr-phosphorylation of ROR2 induced by Wnt5a. *Top*, Phospho-Tyr immunoblot example of HEK-293 cells expressing ROR2 and stimulated for 15 min with 250 ng/ml Wnt5a or nonstimulated (Ctrl). ROR2 was immunoprecipitated and then blotted using an anti-phosphotyrosine antibody. *Bottom* gel, total ROR2 in the samples. *Bottom*, quantification of the intensity of the Phospho-Tyr signal normalized to nonstimulated control present in the same gel (n = 7). Statistical significance was determined using unpaired Student’s *t* test. *Asterisk*, ∗∗ *p* < 0.01. *B*–*E*, Tyr-phosphorylation of ROR2 mutants. *Top*, example gels of Phospho-Tyr immunoblots from HEK-293 cells expressing ROR2 mutants that cannot bind Wnt ligands (*B*; n = 4) or have mutations in the Tyr kinase–like domain (*C*–*E*). Cells were stimulated for 15 min with 250 ng/ml Wnt5a and ROR2 immunoprecipitated. Immunoprecipitate was blotted using an anti-phosphotyrosine antibody. The intensity of the signal was quantified and normalized to nonstimulated control present in the same gel. *Bottom* gels, ROR2 immunoblot of same samples. ROR2-ΔCRD, n = 6; ROR2-ΔTKLD, n = 6; ROR2-K/D3, n = 11; ROR-K507D n = 8. Statistical significance was determined using unpaired Student’s *t* test. CRD, cysteine-rich domain.

We tested whether ROR2 signaling involves tyrosine phosphorylation of the receptor as it is common for other tyrosine kinase receptors that dimerize (41). We expressed recombinant ROR2 in HEK-293 cells and stimulated them with Wnt5a as before. The cells were homogenized, and ROR2 was immunoprecipitated. Immunoblotting of the immunoprecipitate was performed using an anti-phosphotyrosine antibody (EMD Millipore), and the integrated density of the signal was quantified. The membrane was stripped and reprobed with ROR2 antibody to confirm detection of phosphorylated form of ROR2 (see Suplemental Figure). As shown in Figure 2*A*, Wnt5a was found to increase tyrosine phosphorylation of ROR2. This increase in tyrosine phosphorylation is not observed when mutant ROR2-ΔCRD is expressed, confirming that this effect is triggered by the interaction of Wnt5a with the receptor (Fig. 2*B*).

Because of the discrepancy in the literature between structural studies and biochemical studies regarding whether ROR2 has tyrosine kinase activity or not, we tested whether the tyrosine phosphorylation we observed is a result of autophosphorylation. To this end, we created three different mutants that are expected to disrupt the putative kinase activity of ROR2. First, we removed the tyrosine kinase–like domain from ROR2 (residues V473 to W749; ROR2-ΔTKLD). This mutant did not increase its tyrosine phosphorylation when stimulated by Wnt5a (Fig. 2*C*). This could indicate that the receptor has lost its ability to autophosphorylate its tyrosine residues. Alternatively, because there are 12 tyrosine residues in this domain, it is possible that some of those residues are targeted by other kinases present in HEK-293 cells that are activated when ROR2 binds Wnt5a. Additionally, it is reasonable to think that this large domain could also be involved in the allosteric activation of other kinases.

Given that the interpretation of the data with such a large deletion mutant is difficult, we created a single point mutant, ROR2-K507D, and a triple point mutant, ROR2-K/D3, in which lysines 507, 510, and 512 were replaced by aspartate. Lysine 507 is particularly important because it is a conserved lysine that positions the α and β phosphates for catalysis in tyrosine kinases (27, 42). None of these mutants were significantly tyrosine phosphorylated when the receptor was stimulated with Wnt5a (Fig. 2, *D* and *E*), suggesting that an autophosphorylation reaction occurs when the receptor is activated.

Next, we tested whether ROR2 phosphorylation is necessary to activate the downstream cascade that leads to an increase in the trafficking of NMDARs toward synapses. We have reported that Wnt5a activation of ROR2 in neurons and heterologous cells induces an increase in the catalytic activity of PKC and JNK, two downstream kinases that mediate the increase in trafficking of NMDARs (18, 19, 20). To follow in real-time the activation of these kinases, we used CKAR and JNKAR, two FRET-based genetically encoded reporters of PKC and JNK activity, respectively (43, 44).

**Figure 3:**
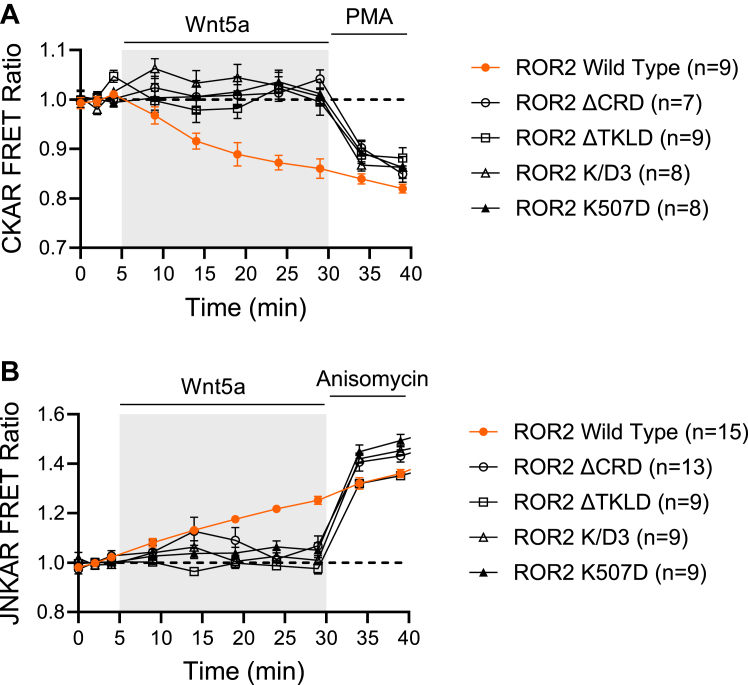
**Wnt5a/ROR2 activation of PKC and JNK.***A* and *B*, optical measurement of PKC (*A*) and JNK (*B*) activity measured as changes in FRET in HEK293 cells co-expressing genetically encoded reporters CKAR or JNKAR and ROR2 WT (*red dots*) or ROR2 mutants that cannot bind Wnt ligands (ROR-ΔCRD) or have mutations in the Tyr kinase–like domain (ROR2-ΔTKLD, ROR2-K/D3, or ROR-K507D). Notice that activation of PKC decreases FRET of CKAR fluorophores, while activation of JNK increases it in JNKAR. Baseline images were taken every 2 min before stimulating with 250 ng/ml Wnt5a. At the end of the experiment, PMA (*A*) or anisomycin (*B*) were used as positive control for PKC and JNK activation, respectively. CRD, cysteine-rich domain; JNK, c-Jun N-terminal kinase; PMA, phorbol 12-myristate 13-acetate.

ROR2 and CKAR were co-expressed in HEK-293 cells, and the receptor activated with Wnt5a. As previously reported in neurons (19), we find that Wnt5a induces activation of PKC in HEK-293 cells as well. FRET efficiency of CKAR decreases when the PKC consensus site between the two fluorophores is phosphorylated (43). Figure 3*A* shows that Wnt5a changes the FRET efficiency of the reporter, indicating activation of PKC. Expression of ROR2-ΔCRD, a mutant that cannot bind Wnt5a, fails to activate PKC, confirming the need for ligand/receptor interaction to activate the signaling cascade. At the end of the experiment, phorbol 12-myristate 13-acetate (PMA) was used as a positive control for PKC activation. Interestingly, mutants in which the putative tyrosine kinase activity of ROR2 has been disrupted, leading to a failure in receptor phosphorylation, also failed to activate PKC (Fig. 3*A*).

Similarly, co-expression of ROR2 with JNKAR was used to follow the activation of JNK kinase upon stimulation of the receptor with Wnt5a (Fig. 3*B*). Notice that FRET efficiency in JNKAR increases when the consensus site for JNK kinase is phosphorylated (44). The mutant that cannot bind Wnt5a, as well as mutants where the putative kinase activity of ROR2 has been disrupted, also failed to activate JNK kinase. In this case, anisomycin was used as a positive control for JNK activation at the end of the experiment.

These experiments support the idea that Wnt5a induces homodimerization of ROR2 to activate an intrinsic tyrosine kinase activity that autophosphorylates the receptor. They also suggest that such tyrosine kinase activity and/or receptor tyrosine phosphorylation is necessary for downstream signaling.

### Chemically induced homodimerization of ROR2

It has been suggested that ROR2 acts as a coreceptor with FZDs, which are classical Wnt receptors involved in Wnt/β-catenin–dependent and Wnt/β-catenin–independent signaling pathways (22, 28, 29, 30).

We tested whether the downstream signaling cascade and the increase in trafficking of NMDARs to synapses could be activated solely by Wnt5a-induced homodimerization of ROR2. To achieve this, we generated a ROR2 mutant that can be induced to homodimerize in the presence of a dimerizing agent. Optically tagged ROR2 receptors were modified by incorporating either FRB or FKBP domains at H101 position in the extracellular domain of the receptor. These domains interact in the presence of rapamycin to form a ternary complex (45), which forces homodimerization of ROR2.

**Figure 4:**
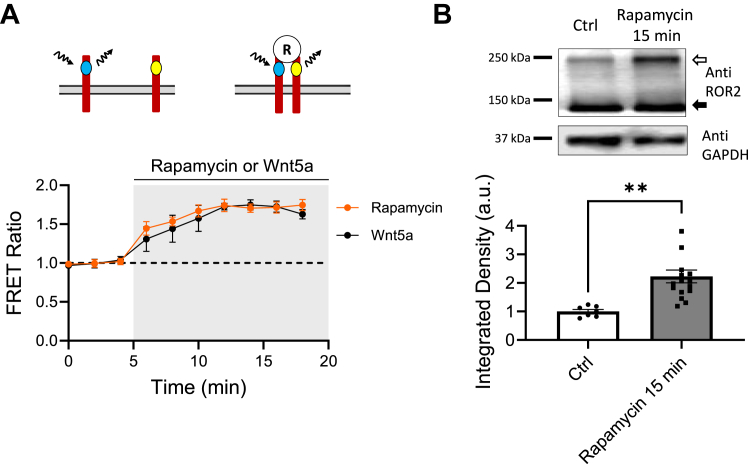
**Chemically induced homodimerization of ROR2.***A*, optical determination of ROR2 homodimerization. FRET ratio of YFP-FKBP-ROR2 and CFP-FRB-ROR2 receptors cotransfected in HEK-293 cells and stimulated with 10 μm rapamycin (*red circles*; n = 10) or 250 ng/ml Wnt5a (*black circles*; n = 10). Z stack images were taken every 2 min during baseline. After stimulation, cells were sampled every 5 min. *B*, biochemical determination of ROR2 homodimerization. *Top*, example of ROR2 immunoblot from HEK-293 cells co-expressing YFP-FKBP-ROR2 and CFP-FRB-ROR2 receptors. Cells were stimulated with 10 μm rapamycin for 15 min (n = 16) and surface proteins crosslinked with BS3 and immunoblotted with a ROR2 antibody. The *open arrow* indicates crosslinked ROR2. *Black arrow* indicates ROR2 monomer. *Bottom* gel, GAPDH immunoblot of same samples. *Bottom*, quantification of the integrated density of the high molecular weight band and normalized to nonstimulated controls (n = 7) present in the same gel. Statistical significance was determined using unpaired Student’s *t* test. *Asterisks*, ∗∗*p* < 0.01.

**Figure 5:**
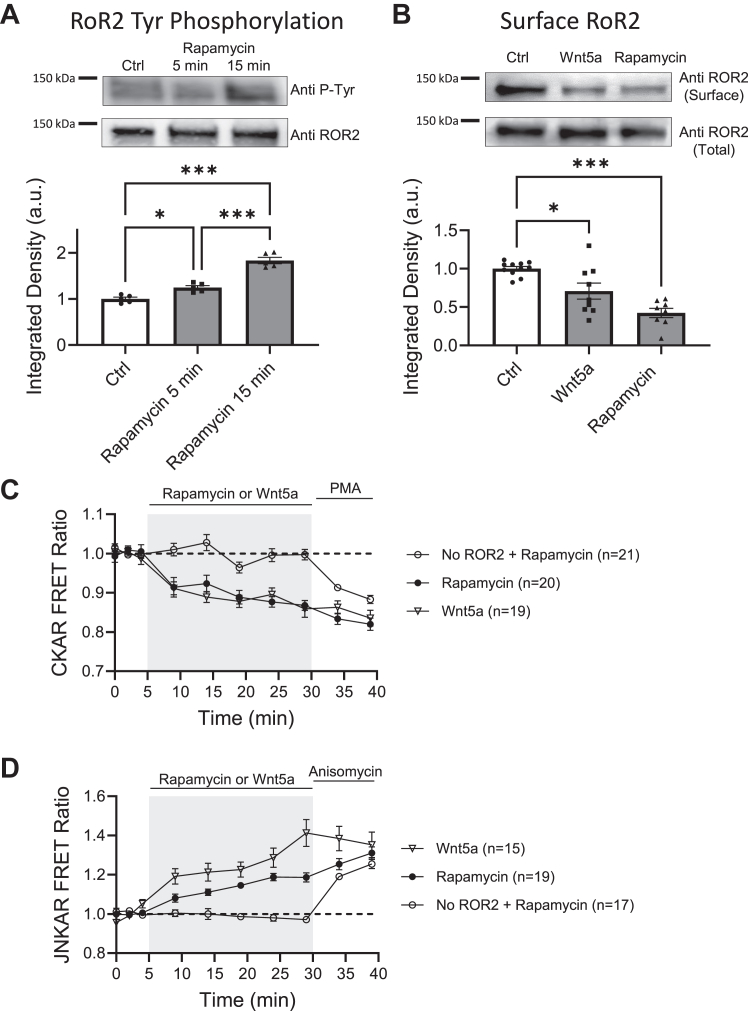
**Homodimerization of ROR2 induces Tyr phosphorylation, internalization of ROR2, and activation of PKC and JNK kinases.***A*, ROR2 Tyr-phosphorylation upon rapamycin-induced homodimerization. *Top*, Phospho-Tyr immunoblot example of HEK-293 cells co-expressing YFP-FKBP-ROR2 and CFP-FRB-ROR2 receptors and stimulated for 5 or 15 min with 10 μm rapamycin or nonstimulated (Ctrl). ROR2 was immunoprecipitated and immunoprecipitate blotted using an anti-phosphotyrosine antibody. *Bottom* gel, total ROR2 in the samples. *Bottom*, quantification of the integrated intensity of the Phospho-Tyr signal normalized to nonstimulated control present in the same gel (n = 5). Statistical significance was determined using one-way ANOVA (F = 69.78; *p* < 0.001) and Tukey’s multiple comparisons test. *Asterisks*, ∗*p* < 0.05, ∗∗*p* < 0.01, ∗∗∗*p* < 0.001. *B*, biotinylation of surface ROR2. *Top*, example of ROR2 immunoblot from HEK-293 cells co-expressing YFP-FKBP-ROR2 and CFP-FRB-ROR2 receptors and stimulated with 250 ng/ml Wnt5a or 10 μm rapamycin. Surface proteins of cells co-expressing rapamycin-sensitive ROR2 receptors were biotinylated and immunoprecipitated with avidin-agarose. The immunoprecipitate was then immunoblotted using an ROR2 antibody. *Bottom* gel, total ROR2 in the samples. *Bottom*, quantification of ROR2 surface expression after stimulation with 250 ng/ml Wnt5a (n = 9) or 10 μm rapamycin (n = 8). ROR2 signal was quantified and expressed as percentage of nonstimulated cells (n = 11) present in the same gel. Statistical significance was determined using one-way ANOVA (F = 16.47; *p* < 0.001) and Tukey’s multiple comparisons test. *Asterisks*, ∗*p* < 0.05, ∗∗*p* < 0.01, ∗∗∗*p* < 0.001. *C* and *D*, optical measurement of PKC (*C*) and JNK (*D*) activity measured as changes in FRET in HEK293 cells co-expressing genetically encoded reporters CKAR or JNKAR and rapamycin-sensitive ROR2 receptors. Cells were stimulated with 250 ng/ml Wnt5a or 10 μm rapamycin. Baseline images were taken every 2 min before stimulation as indicated. At the end of the experiment, PMA (C) or anisomycin (*D*) were used as positive control for PKC and JNK activation, respectively. JNK, c-Jun N-terminal kinase.

We co-express rapamycin-sensitive ROR2 receptors tagged with CFP (CFP-FRB-ROR2) and YFP (YFP-FKPB-ROR2) in HEK-293 cells to measure homodimerization using FRET. Upon addition to the bath, rapamycin increases FRET signal at a similar level and with similar kinetics as Wnt5a. This demonstrates that rapamycin can induce homodimerization of ROR2, as shown by the increase in FRET signal (Fig. 4*A*). To confirm this result, we crosslinked surface proteins as before (Fig. 1*B*) and determined the proportion of crosslinked ROR2, indicated by a doubling in the molecular weight of ROR2. Indeed, rapamycin increased the amount of ROR2 receptor detected at around 250 KD (Fig. 4*B*). ROR2 homodimerization induced by rapamycin also increased the level of tyrosine phosphorylation detected in ROR2, as shown in Figure 5*A*. We also observed that activation of ROR2 by Wnt5a resulted in some internalization of the receptor after 15 min, as has been previously reported (28, 46). After activation of the receptor, the surface proteins were biotinylated, precipitated with avidin-agarose, and immunoblotted with an anti-ROR2 antibody. Wnt5a caused a decrease in the amount of ROR2 on the cell surface (Fig. 5*B*). Similar results were obtained with rapamycin-sensitive ROR2 receptors in response to rapamycin (Fig. 5*B*).

Together, these results indicate that rapamycin induces ROR2 homodimerization, increases its level of tyrosine phosphorylation, and triggers a ligand-induced internalization of the receptor.

### Homodimerization of ROR2 is sufficient to activate PKC and JNK

Using rapamycin-sensitive ROR2 receptors, we analyzed whether ROR2 homodimerization is sufficient to activate the neuronal Wnt signaling pathway that regulates the synaptic incorporation of NMDA-type glutamate receptors. Specifically, we focused on key steps of the signaling cascade (20). We tested the ability of rapamycin-sensitive ROR2 receptors to activate PKC and JNK kinases, increase somatic and dendritic Ca^2+^ levels, and enhance surface and synaptic expression of NMDARs.

HEK-293 cells were cotransfected with FRB-ROR2 and FKPB-ROR2, the rapamycin-sensitive receptors, along with either CKAR, the genetically encoded activity reporter of PKC, or JNKAR, the genetically encoded activity reporter of JNK. Figure 5*C* shows that rapamycin induces PKC activation to levels and with kinetics similar to those of Wnt5a. To test whether rapamycin directly activates PKC, cells expressing only CKAR were used as controls. Rapamycin-induced PKC activation relies on ROR2 receptors, as it activated PKC only in cells expressing ROR2 receptors (Fig. 5*C*). Similarly, rapamycin activated JNK kinase in an ROR2-dependent manner (Fig. 5*D*). These results indicate that ROR2 homodimerization is sufficient to activate PKC and JNK kinases.

**Figure 6:**
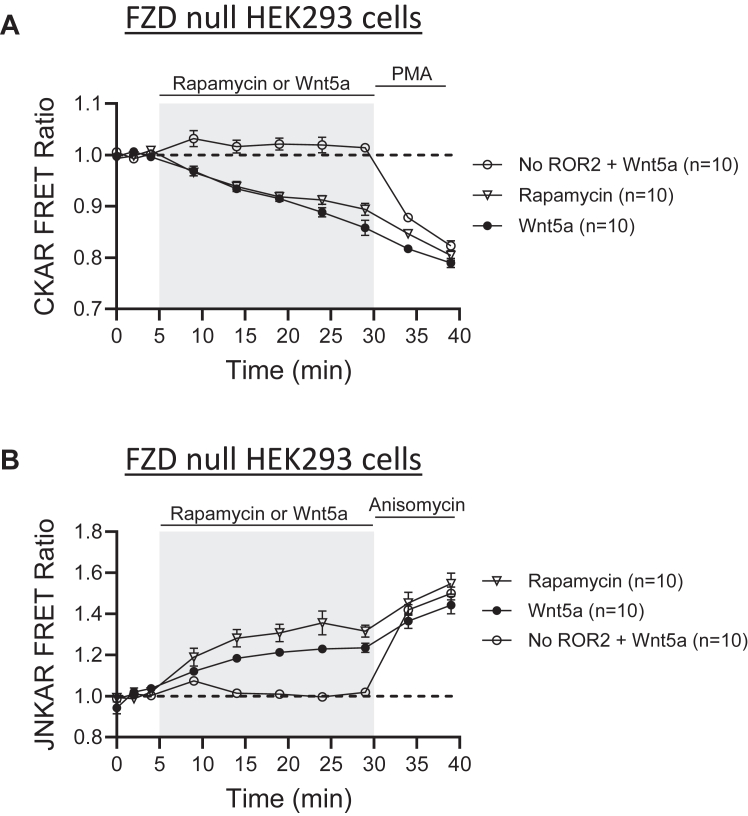
**ROR2 activation of PKC and JNK kinases in FZD null HEK-293 cells.***A* and *B*, optical measurement of PKC (*A*) and JNK (*B*) activity measured as changes in FRET in HEK-293 FZD1-10 KO cells co-expressing genetically encoded reporters CKAR or JNKAR and rapamycin-sensitive ROR2 receptors. Cells were stimulated with 250 ng/ml Wnt5a or 10 μm rapamycin. Baseline images were taken every 2 min before stimulation as indicated. At the end of the experiment, PMA (*A*) or anisomycin (*B*) were used as positive control for PKC and JNK activation, respectively. PMA, phorbol 12-myristate 13-acetate; JNK, c-Jun N-terminal kinase; FZD, Frizzled receptor.

Given that FZDs are expressed in both HEK293 and primary neurons, it is always challenging to completely exclude their potential contribution to the studied signaling cascade. To rule out possible contributions of endogenous FZDs, we repeated these experiments in HEK-293 cells where all ten FZDs have been knockout (Fig. 6) (47). HEK-293 FZD1-10 KO cells were cotransfected with CKAR or JNKAR along with rapamycin-sensitive receptors. In these cells, just like in regular HEK-293 cells, Wnt5a and rapamycin were able to activate PKC (Fig. 6*A*) and JNK (Fig. 6*B*). Importantly, cells without recombinant ROR2 expressed did not respond to Wnt5a.

The use of rapamycin-sensitive receptors and the fact that activation of PKC and JNK still occurs in the absence of FZDs provide compelling evidence that Wnt5a and ROR2 can signal in an FZD-independent manner.

### Homodimerization of ROR2 in neurons is sufficient to activate the Wnt/Ca^2+^ signaling cascade

**Figure 7:**
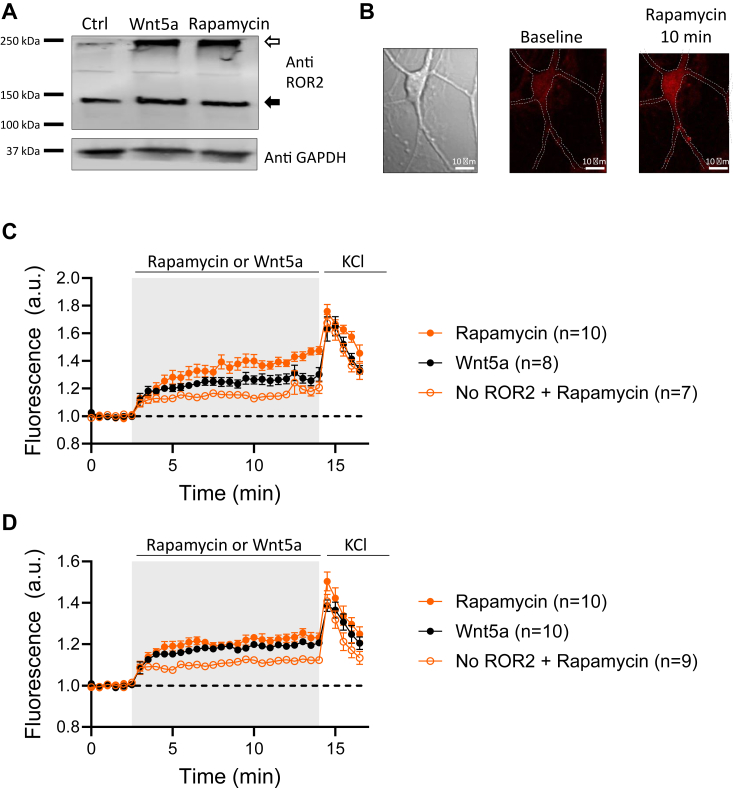
**Homodimerization of ROR2 elevates intracellular Ca**^**2+**^**in neurons.***A*, biochemical determination of ROR2 homodimerization in neurons. Dissociated cultured neurons co-expressing YFP-FKBP-ROR2 and CFP-FRB-ROR2 receptors were stimulated with 10 μm rapamycin or 250 ng/ml Wnt5a for 15 min and surface proteins crosslinked with BS3 and immunoblotted with an ROR2 antibody. The *open arrow* indicates crosslinked ROR2. *Black arrow* indicates ROR2 monomer. *Bottom* gel, GAPDH immunoblot of same samples. *B*, sample images of a cultured neuron loaded with fluorescent Ca^2+^ indicator and expressing rapamycin-sensitive ROR2 receptors before and after stimulation with 10 μm rapamycin for 10 min. *C* and *D*, quantification of intracellular Ca^2+^ at the soma (*C*) or dendrites (*D*) of cultured dissociated neurons cotransfected with YFP-FKBP-ROR2 and CFP-FRB-ROR2. Neurons were loaded with Ca^2+^ indicator and stimulated either with 250 ng/ml Wnt5a or 10 μm rapamycin. At the end of the experiment, KCl was added as a positive control.

We transfected dissociated cultured neurons with rapamycin-sensitive ROR2 receptors to test the physiological relevance of ROR2 homodimerization in neurons. First, we tested the ability of rapamycin to induce ROR2 homodimerization in neurons using the crosslinking assay described earlier. Figure 7*A* shows that stimulation of neurons with either Wnt5a or rapamycin increases the proportion of ROR2 detected at ∼250 KD, double the normal size of ROR2, indicating homodimerization of the receptor.

Elevation of intracellular Ca^2+^ is necessary for Wnt5a-induced trafficking of NMDARs to the surface of neurons and their subsequent insertion into functional synapses. This Wnt5a/Ca^2+^ signaling in neurons is ROR2-dependent and requires activation of voltage-gated Ca^2+^ channels as well as Ca^2+^ release from intracellular stores (18, 20). To test whether homodimerization of ROR2 is sufficient to induce a Ca^2+^ increase in the soma and/or dendrites of neurons, cultured dissociated neurons were cotransfected with FRB-ROR2 and FKPB-ROR2 and loaded with Ca^2+^ indicator Biotracker 609. After 3 min of baseline, rapamycin was added to the bath. Rapamycin increased somatic Ca^2+^ as well as dendritic Ca^2+^ similarly to the effect produced by Wnt5a (Fig. 7, *B*–*D*). At the end of the experiment, neurons were depolarized with KCl as a positive control. Interestingly, neurons that did not express rapamycin-sensitive ROR2 receptors also responded to rapamycin by elevating intracellular Ca^2+^. Nevertheless, the presence of rapamycin-sensitive receptors produced a more robust Ca^2+^ increase (Fig. 7, *C* and *D*). These experiments indicate that chemically induced homodimerization of ROR2 is sufficient to increase intracellular Ca^2+^ in neurons.

To determine whether ROR2 homodimerization is sufficient to enhance NMDAR trafficking, we evaluated the surface expression and functional incorporation of NMDARs into synapses. Wnt5a increases surface expression of GluN2B-containing NMDARs and their synaptic incorporation on neurons. This leads to an increase in the amplitude of NMDAR-mediated excitatory postsynaptic currents (EPSCs) and lowers the threshold for synaptic plasticity (18, 19, 20). GluN2B-containing receptors are readily mobile in the membrane of neurons (48, 49, 50) and, when incorporated into synapses, critical for the induction of synaptic plasticity (51).

**Figure 8:**
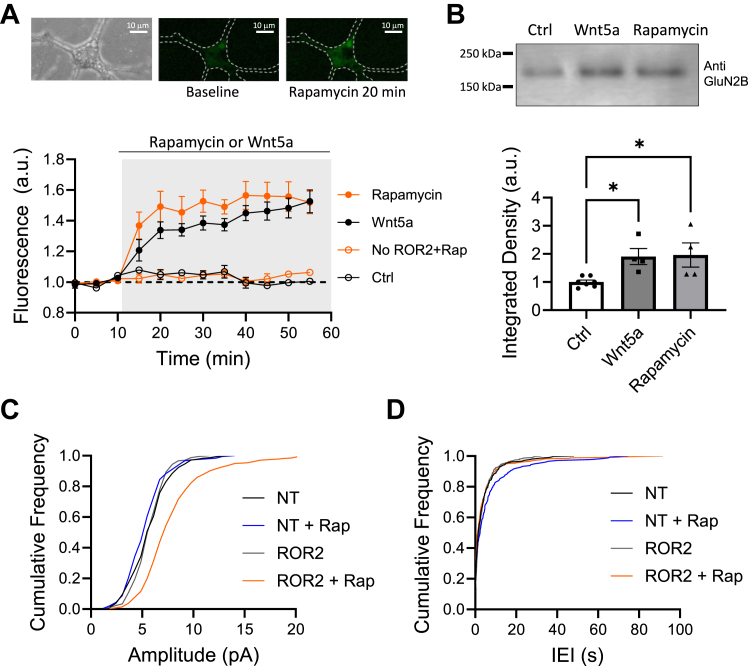
**Homodimerization of ROR2 is sufficient to increase trafficking of NMDARs in neurons.***A*, optical detection of surface GluN2B. *Top*, sample images of a cultured neuron co-expressing rapamycin-sensitive ROR2 receptors and SEP-GluN2B before and after rapamycin stimulation. *Bottom*, quantification of fluorescence in the cell body of neurons co-expressing YFP-FKBP-ROR2, CFP-FRB-ROR2, and SEP-GluN2B stimulated with either 10 μm rapamycin (n = 7), 250 ng/ml Wnt5a (n = 9), or control cells nonstimulated (n = 3). *Open red circles* are cells expressing only SEP-GluN2B and stimulated with rapamycin (No ROR2 + rap; n = 8). *B*, biotinylation of surface endogenous GluN2B. *Top*, example of GluN2B immunoblot from neurons co-expressing mCherry-FRB-ROR2 and mCherry-FKPB-ROR and stimulated with 250 ng/ml Wnt5a or 10 μm rapamycin. Surface proteins of neurons co-expressing rapamycin-sensitive ROR2 receptors were biotinylated and immunoprecipitated with avidin-agarose. The immunoprecipitate was then immunoblotted using a GluN2B antibody. *Bottom*, quantification of GluN2B immunoblots from neurons co-expressing rapamycin-sensitive receptors and stimulated either with Wnt5a (n = 5), rapamycin (n = 5), or control nonstimulated neurons (n = 7). Statistical significance was determined using one-way ANOVA (F = 5.758; *p* < 0.05) and Tukey’s multiple comparisons test. *Asterisk*, ∗*p* < 0.05. *C*, cumulative frequency distribution of the amplitude of NMDAR-mediated mEPSCs. Voltage clamp recordings were obtained at −40 mV in the presence of TTX (1 mM) and NBQX (2 mM) from two groups: nontransfected neurons (NT) and neurons transfected with rapamycin-sensitive ROR2 receptors (ROR2). Prior to recording mEPSCs, neurons were either stimulated with 10 μM rapamycin for 15 min or left unstimulated. Neurons were prepared from 3 to 4 p1 pups and a minimum of six different preparations were used. The number of cells recorded, and total number of events scored are as follows: NT = 6 neurons, 314 events; NT + Rap = 6 neurons, 181 events; ROR2 = 6 neurons, 300 events; ROR2 + Rap = 9 neurons, 414 events. *D*, cumulative frequency distribution of the inter event interval (IEI) of NMDAR-mediated mEPSCs. Cumulative probability plots of mEPSC IEI from whole-cell recordings as in (*C*). mEPSC, miniature EPSC; NMDAR, NMDA-type glutamate receptor; SEP, super ecliptic phluorin.

Cultured dissociated neurons were cotransfected with rapamycin-sensitive ROR2 receptors, GluN1, and GluN2B optically tagged with super ecliptic phluorin (SEP), which is a pH sensitive form of GFP (52). The SEP tagging of the GluN2 subunit enabled us to monitor the surface expression of NMDARs containing GluN2B. Cotransfection of the GluN1 subunit is necessary to form a functional recombinant receptor with the GluN2B subunit, enabling proper trafficking of the receptor towards the plasma membrane and its synaptic expression (53). We measured changes in SEP fluorescence after addition of rapamycin to the bath (Fig. 8*A*, top). Figure 8*A*, bottom, shows that rapamycin, like Wnt5a, induced an increase in SEP fluorescence measured in the soma of neurons, indicating incorporation of GluN2B-containing NMDARs into the plasma membrane. The effect of rapamycin was ROR2-dependent as GluN2B surface fluorescence did not change in neurons where rapamycin-sensitive receptors were not expressed.

We also employed a biochemical assay to examine the surface expression of GluN2B-containing NMDARs. Specifically, we biotinylated the surface proteins of cultured-dissociated neurons that had been cotransfected with rapamycin-sensitive ROR2 receptors, GluN1, and GluN2B. Neurons were stimulated either with Wnt5a or rapamycin for 15 min. Following protein precipitation with avidin-agarose, samples were immunoblotted with an anti GluN2B antibody, and the intensity of the band was quantified and normalized to control samples. This allowed for the determination of the amount of surface expression of GluN2B-containing NMDARs. As shown in Figure 8*B*, Wnt5a as well as rapamycin increased the number of GluN2B-containing NMDARs present in the plasma membrane.

To measure the functional incorporation of NMDARs into synapses, we recorded the amplitude of miniature spontaneous EPSCs (mEPSCs) mediated by NMDARs in cultured-dissociated neurons in the presence of tetrodotoxin. Spontaneous activity was measured in voltage clamp configuration at a holding potential of −40 mV to relieve the Mg^2+^ blockade of the receptor and to maximize the detection of NMDAR-mediated currents. NBQX, a selective antagonist of AMPA-type glutamate receptors, was used to block the contribution of AMPA receptors to the measured currents. Neurons transfected with rapamycin-sensitive ROR2 and treated with rapamycin for 15 min exhibited mEPSCs with a significantly larger amplitude, consistent with a larger number of NMDARs at synapses (Fig. 8*C*). Inter-event interval of mEPSCs was not altered, indicating there has been no change in the total number of synapses (54) because of rapamycin treatment (Fig. 8*D*).

All together, these results indicate that the chemically induced ROR2 homodimerization triggers an increase in intracellular Ca^2+^ in neurons, activates the kinases involved in the trafficking of NMDARs, and enhances the transport of GluN2B-containing NMDARs to the neuronal surface, leading to their functional integration into synapses.

## Discussion

In this study, we aimed to better understand the mechanism of ROR2 activation and signaling, which leads to activation of the neuronal Wnt5a/Ca^2+^ pathway. This pathway regulates the trafficking of NMDA-type glutamate receptors toward synapses, thereby affecting the threshold for synaptic plasticity, which serves as a cellular model of learning and memory. Furthermore, the neuronal Wnt5a/Ca^2+^ pathway increases the excitability of neurons by affecting their intrinsic properties. These effects on synaptic and neuronal function are dependent on ROR2 (18, 19, 20).

Measurements of FRET provide direct evidence that Wnt5a induces homodimerization of ROR2. While this approach cannot establish stoichiometry, it indicates that Wnt5a can bring at least two ROR2 molecules into proximity. This finding is consistent not only with the structural similarity between ROR2 and tyrosine kinase receptors, which also dimerize upon ligand binding (41), but also with previous biochemical reports (25, 35) and our own biochemical experiments (Figs. 1*B* and 7*A*). The Wnt5a-induced homodimerization of ROR2 requires the presence of the CRD, which acts as a ligand-binding site in all FZD and ROR receptors. While the exact mechanism of how one Wnt can interact with two receptors simultaneously is not fully understood, it has been shown that Wnts can induce the formation of complexes involving FZD receptors and either the coreceptor LRP5/6 or ROR2 (29, 55). A conserved set of residues from discontinuous regions of the primary structure of Wnts has been proposed as a binding site, which could enable the formation of ternary complexes. They form a patch that lies in the surface of Wnts, which is located opposite to the end that binds the CRD domain of Wnt receptors. In the crystal structure of an interacting Wnt and an FZD receptor, this patch interfaces between two Wnt/FZD pairs, resulting in the formation of an asymmetric Wnt/FZD dimer (56). Whether a similar model could explain the homodimerization of ROR2 induced by Wnt5a remains to be investigated. Nonconserved amino acid substitutions in the patch involved in the formation of the asymmetric Wnt/FZD dimer could provide some level of specificity for Wnt5a in the homodimerization of ROR2, compared to other Wnts involved in β-catenin–dependent pathways (56, 57).

Hippocampal neurons from juvenile mice express several receptors from the Frizzled family (31, 32, 33, 34); thus, it is plausible that even though ROR2 is capable of homodimerization, the activation of the neuronal Wnt5a/Ca^2+^ pathway by Wnt5a occurs through the formation of FZD/ROR2 heterodimers, where ROR2 is only necessary but not sufficient. To determine whether ROR2 alone is capable of activating the neuronal Wnt5a/Ca^2+^ cascade, we engineered ROR2 mutants that are sensitive to rapamycin. We show that rapamycin brings optically tagged ROR2 receptors together, resulting in an increase in the FRET ratio of the fluorophores. More importantly, chemically induced homodimerization alone was sufficient to increase the activity of PKC and JNK kinases, increase both somatic and dendritic Ca^2+^, and enhance surface expression of GluN2B-containing NMDARs, as well as the number of synaptic NMDARs. To control for unintended effects of rapamycin, we repeated the experiments in cells that had not been transfected with the rapamycin-sensitive ROR2 receptors. By itself, rapamycin did not show any effects in our measurements of PKC or JNK activity nor in the trafficking of NMDARs. However, it did increase somatic and dendritic Ca^2+^ in nontransfected neurons, albeit to a lesser extent than when the rapamycin-sensitive receptors were expressed. This suggests that rapamycin and ROR2 homodimerization elicit distinct mechanisms for cytosolic Ca^2+^ elevation that are not mutually exclusive. This finding is consistent with the lack of effect of rapamycin alone on the downstream signaling cascade. It suggests that the location and manner of Ca^2+^ elevation play a crucial role in decoding the signal triggered by ROR2 homodimerization. To further support the idea that ROR2 homodimerization alone is capable of initiating the neuronal Wnt5a signaling cascade, we demonstrate that the activation of ROR2, induced by either Wnt5a or rapamycin, results in an elevation of PKC and JNK activity, even when FZDs are absent in HEK-293 FZD1-10 KO cells. Together, our results indicate that in neurons, ROR2 can activate signaling pathways independently of FZDs by forming homodimers. Nonetheless, it is possible that ROR2 could act as a coreceptor in certain contexts with FZDs in neurons. Future studies should address the potential role of ROR2/FZDs heterodimerization in the functioning of neurons.

The ROR2 receptor has been classified as a pseudokinase due to amino acid substitutions in conserved motifs common to other receptor tyrosine kinases (27, 58). Additionally, the crystalized conformation of ROR2 TKD closely resembles that of the inactive insulin receptor kinase (27, 59). We observed an increase in tyrosine phosphorylation of ROR2 upon dimerization, either through Wnt5a or rapamycin. This suggests that, if ROR2 is truly a pseudokinase, another signaling molecule may be allosterically regulated either already bound to the receptor or recruited upon homodimerization. To shed light on this issue, we mutated K507 in the conserved VAIK motif of ROR2. This conserved lysine typically positions the ATP alpha and beta phosphates for catalysis (42). However, in ROR2, it appears to be situated at a distance from the ATP phosphates, which would prevent it from contributing to the reaction (27). Nevertheless, substitution of K507 with aspartate prevented the previously observed increase in tyrosine phosphorylation of the receptor. Importantly, this substitution also prevented activation of downstream effectors such as PKC and JNK. Similar results were obtained with a triple mutant K507, 510, 512D, as well as a ROR2 lacking the entire TKD. These results are consistent with previous biochemical reports that indicate that K507 mutant of ROR2 affects not only its own phosphorylation but also the phosphorylation of other substrates (24). While it is possible that this point mutation alters the interaction between ROR2 and another signaling molecule that is either already associated with the receptor or recruited to the homodimer, a more parsimonious explanation is that ROR2 exhibits some tyrosine kinase activity. This activity could initiate the cascade *via* a trans-autophosphorylation event of the homodimer partner. This explanation does not rule out the possibility that other kinases could become involved in a second step. It is important to remark that the structure of ROR2 exhibits the common kinase topology, in which the crucial motifs identified in other tyrosine kinases are conserved or slightly modified. Specifically, the activation loop YxxxYY and the previously mentioned VAIK motif are conserved. The HRD motif, which normally contributes the catalytic aspartic acid residue, presents a conserved substitution of arginine to lysine in ROR2. A more substantial substitution occurs in the DFG motif, where the phenylalanine is replaced by a leucine in ROR2. These two residues, the arginine and phenylalanine, are conserved in most kinases but not in all, which opens the possibility that these substitutions do not prevent ROR2 from catalyzing a phosphorylation reaction. Using a thermal shift assay, it has been shown that ROR1 does not bind ATP (42). Although the amino acid sequences of ROR1 and ROR2 exhibit only small differences, it would be important to determine if the same occurs with ROR2. Future studies to identify the phosphorylated tyrosine residues and elucidate how ROR2 connects with downstream effectors will enhance our understanding of ROR2's activation mechanism.

Our discovery that ROR2 can initiate signaling independently of FZD receptors has significant implications for our understanding of cellular communication and its roles in health and disease. ROR2 is highly expressed in the hippocampus with minimal expression in other brain regions (19, 31). This observation opens possibilities for the development of targeted therapies aimed at regulating hippocampal function without affecting other pathways where FZDs are involved. Additionally, it has relevance to various cancer types where ROR2 plays a role. Our findings suggest that receptors within the Wnt pathways can have distinct functions and activation modes. Future investigations may need to consider the individual roles of receptors like ROR2 in specific cellular contexts.

## Experimental procedures

### Cell cultures

HEK-293 cells were cultivated in Dulbecco’s modified Eagle’s medium (Gibco) supplemented with 10% fetal bovine serum (Sigma-Aldrich) and 2% penicillin/streptomycin (Gibco) and used between passages 10 and 35. HEK-293 cells lacking all FZDs (FZD1-10 KO cells) were kindly provided by Dr Benoit Vanhollebeke (47).

Dissociated hippocampal neurons cultures were prepared from hippocampi collected from mice CB57BL/6G of both sexes at postnatal day 1 (p1). Tissue from 3 to 4 animals was collected in cold dissection medium containing Hanks’ Plus HBBSS+, 10 mM Hepes, 33.3 mM glucose, 0.3% bovine serum albumin, and 12 mM MgSO_4_, processed, and neurons plated at a density of 200,000 cells per 35 mm plates. Neurons were cultured in neuronal medium containing MEM, 25 mM Hepes, 20 mM glucose, 10% horse serum, 2% B27, 1% sodium pyruvate, GlutaMax, and 1% penicillin/streptomycin. After 6 days *in vitro*, neurons were supplemented with 20 μM fluorodeoxyuridine. Neurons were used between 12 and 18 days *in vitro*. HEK-293 cells were transfected at 75 to 85% confluency using Lipofectamine LTX (Invitrogen) and constructs expressed for 24 to 36 h. Neurons were transfected using lipofectamine 2000 (Invitrogen), and constructs expressed for 24 to 36 h.

When indicated, cells were stimulated with either 250 ng/ml of recombinant Wnt5a (R&D Systems, 645-WN-010) or 10 μm rapamycin (LC Labs, R-5000).

### DNA plasmids and constructs

All ROR2 constructs used are from *Rattus norvegicus*, accession number NM_001107339. Optically tagged ROR2 receptors and ROR2 lacking the CRD domain have been reported previously (19). Briefly ROR2-mCherry, CFP-ROR2, and YFP-ROR2 were generated by inserting the fluorescent protein after E35 in the amino terminal domain of rat ROR2. ROR2-mCherry ΔCRD was generated by removing amino acids R178 through Y313. We generated ROR2-ΔTKL, a mutant lacking the tyrosine kinase-like domain, by removing amino acids V473 to W749. ROR2-K/D3, a triple point mutant, was generated by mutating Lys 507, 510, and 512 into Asp.

Genetically encoding probe CKAR (43) was obtained from Addgene, plasmid 14860. JNKAR was obtained from Dr Jin Zhang (44).

Chemically induced homodimerizable ROR2 receptors were generated by inserting either the FRB domain of mTOR or the rapamycin-binding domain of FKBP. Both sequences were inserted at position H101 of optically tagged ROR2 receptors to generate FRB-ROR2 and FKPB-ROR2 tagged with either CFP, YFP, or mCherry.

### PKC and JNK activity assays

*In vivo* kinase activity of PKC and JNK was determined using a FRET assay using genetically encoded kinase activity reporters CKAR or JNKAR. HEK-293 cells were cotransfected with different optically tagged ROR2 receptors and either CKAR or JNKAR, genetically encoded probes for PKC and JNK respectively (43, 44). The link between the two fluorophores in the probes, FRET pair, contains a phosphorylation consensus site for either PKC (CKAR) or JNK (JNKAR). Activation of the kinases and subsequent phosphorylation of the reporters changes FRET efficiency, allowing *in vivo* real time imaging of PKC or JNK activation. Notice that phosphorylation of CKAR by PKC decreases FRET (43), while phosphorylation of JNKAR by JNK increases it (44).

Constructs were expressed 24 to 36 h before imaging using a Zeiss LSM 710 confocal microscope with a 40X water immersion objective. An excitation laser at 458 nm and a set of filters to collect emitted light between 514 to 530 nm were used. Baseline images were captured every 2 min, and cells were stimulated either with Wnt5a (250 ng/ml) or rapamycin (10 μM), with images collected every 5 min. At the end of the experiment, cells were stimulated with either PMA (1 μM) or anisomycin (10 μM) as positive controls.

### Antibodies and immunoblots

Cells were homogenized in 500 μl of lysis buffer containing 50 mM Tris (pH = 7.4), 150 mM NaCl, 5 mM EDTA, 1 mM EGTA, 0.5 g protease inhibitor cocktail (Thermo Fisher Scientific), 10% Triton X-100, 0.01 g deoxycholate, and centrifuged at 10,000 rpm for 10 min. Supernatant was collected and proteins quantified using the bovine serum albumin method. Equal amounts of protein per sample were separated in a 7.5% or a gradient 4 to 15% SDS-PAGE (Bio-Rad) and transferred to a nitrocellulose membrane for immunoblotting.

Anti-ROR2 antibody from DSHB (AB_10804796) was used at a dilution of 1:5000. Specificity of the antibody was established by immunoblot of samples from untransfected HEK-293 and ROR2 transfected cells (Suplemental Figure*A*). The same antibody was used to immunoprecipitate ROR2. The specificity of the immunoprecipitation was established by comparing the amount of ROR2 in the immunoprecipitate from immunoprecipitations using a control IgG or ROR2 antibody (Suplemental Figure*C*). Anti-GluN2B was obtained from Santa Cruz (sc-365597). Anti-phosphotyrosine antibody was from EMD Millipore (05-321).

Chemiluminescence signal (SuperSignal West Femto, Thermo Fisher Scientific) was captured using a C-DiGit Blot Scanner (LI-COR Biosciences, USA) and quantified with Image-J.

### Crosslinking and biotinylation assays

HEK-293 cells or neurons were incubated for 45 s with crosslinking reagent BS3 (Pierce PG82083) and the reaction stopped with 100 mM Tris. Cells were washed 3 times with cold PBS, homogenized, and processed for immunoblotting of ROR2.

To estimate surface proteins, HEK-293 cells were incubated at 4 °C for 30 min with biotin (0.5 mg/ml) protected from light. The reaction was stopped with 100 mM Tris and cells washed 3 times with cold PBS. Cell lysates were then incubated with Avidin Agarose (Pierce, 20225), centrifuged at 10,000 rpm for 2 min, and proteins immunoblotted for ROR2 or GluN2B.

### Fluorescence imaging

A Zeiss710 confocal microscope was used to measure FRET at room temperature in cells expressing proteins of interest. Z-stack images were obtained every 5 min using 458 nm excitation. Light emitted by excited fluorophore was detected and measured at 530 nm.

Corrected total cell fluorescence (CTCF) for each channel was calculated as cell integrated density − (area of cell integrated density ∗ mean background). FRET ratio was calculated as YFP CTCF divided by CFP CTCF. FRET ratios were normalized to the average of baseline (19).

Calcium imaging was performed in cultured dissociated neurons loaded with 2 μM calcium indicator Biotracker 609 red Dye (EMD Millipore). Z-stack images were acquired every 30 s. After 15 min, 50 mM KCl was added as positive control of neuronal response.

Detection of SEP-tagged GluN2B subunits of the NMDAR was done in cultured dissociated neurons obtained from 3 to 4 p1 animals and cotransfected with rapamycin-sensitive ROR2, SEP-GluN2B SEP, and GluN1 subunits. Cell body fluorescence Z-stacks were taken every 5 min. One cell per dish was imaged and experiments were repeated in neurons coming from at least eight different preparations. Fluorescence images were analyzed using Image-J (https://imagej.net/) and fluorescence values normalized to the average of baseline images.

### Electrophysiology

Recordings of spontaneous NMDA-mediated events were made from neurons in dissociated cultures at 11 to 14 days in culture. They were recorded in a modified ACSF (124 mM NaCl; 2.5 mM KCl; 1.2 mM NaH_2_PO_4_; 24 mM Hepes; 12.5 mM glucose; 2 mM CaCl_2_; 2 mM MgSO_4_) at 30 to 32 °C. Tetrodotoxin (Tocris, 1 μM), Picrotoxin (Tocris, 100 μM), and NBQX (Tocris, 20 μM) were bath applied. Whole-cell voltage clamp recordings were made using 3 to 5 MOhm electrodes containing a cesium-based internal solution (115 mM CsMeO_4_; 20 mM CsCl; 2.5 mM MgCl_2_; 10 mM Hepes; 4 mM ATP; 0.4 mM GTP; 10 mM Na-Phosphocreatine; 0.6 mM EGTA). Following break in, cells were held at −40 mV, and spontaneous activity was recorded for 5 to 10 min. mCherry-ROR2–transfected cells were identified using an epifluorescence lamp. In recordings in which rapamycin was used, 10 μM rapamycin was added to the bath and allowed to circulate for a minimum of 15 min prior to cell patching and recording. Miniature events were identified and analyzed using MiniAnalysis (Synaptosoft).

### Statistics

Mean values are given with s.e.m. *p*-values, and statistical significance were determined using one-way ANOVA with Tukey’s multiple comparisons test for three or more groups. Cumulative distributions of frequency and amplitude data from mEPSCs was analyzed using Kolmogorov-Smirnov test. F and *p* values for the ANOVA tests and *p* values for post hoc test are provided in the legend of the corresponding figure.

## Data availability

All the data described in the manuscript are contained within the manuscript.

## Supporting information

This article contains supporting information.

## Conflict of interest

The authors declare that they have no conflicts of interest with the contents of this article.

## Supporting information

Supplemental figures *A*, anti-ROR2 antibody control. ROR2 immunoblot of HEK293 cells and HEK293 cells transfected with ROR2. A specific band of the right molecular weight was detected only on HEK-293 cells transfected with recombinant ROR2. *B*, anti-phosphotyrosine antibody control. HEK293 cells were transfected with ROR2 and stimulated for 15 min with 250 ng/ml Wnt5a. Cells were lysed in the absence or in the presence of a phosphatase inhibitor cocktail. ROR2 was immunoprecipitated from the cell lysate with anti-ROR2 antibody. The immunoprecipitate was then immunoblotted using a Phospho-Tyr–specific antibody. After phosphotyrosine detection, the membrane was stripped and reblotted with anti-ROR2 antibody to verify the target protein phosphorylation. *C*, ROR2 immunoprecipitation control. HEK293 cells expressing ROR2 were lysed and immunoprecipitated either with a control IgG or anti-ROR2 antibody. The immunoprecipitated was separated by SDS-PAGE and immunoblotted with a ROR2 antibody.
