# Supplementary figures and images for: ROR2 homodimerization is sufficient to activate a neuronal Wnt/calcium signaling pathway
Source: J Biol Chem. 2023 Oct 12;299(11):105350. doi: 10.1016/j.jbc.2023.105350 (PMC10654037; doi:10.1016/j.jbc.2023.105350)

**A**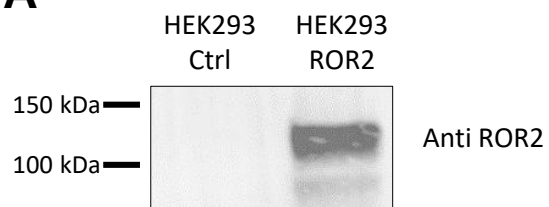**B**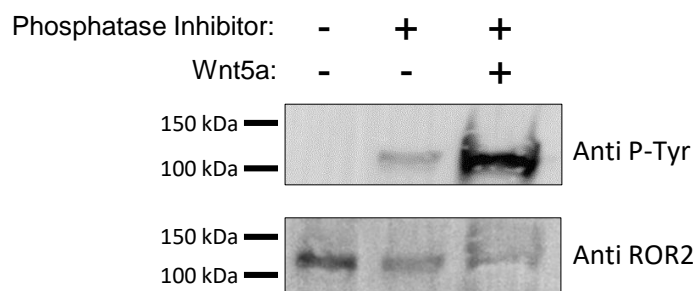**C**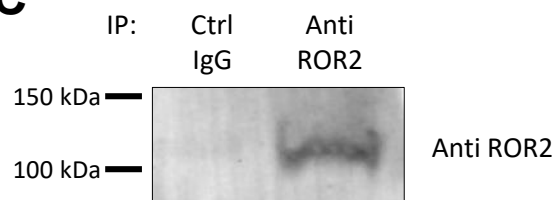

Supplement: Supplemental figures — A, anti-ROR2 antibody control. ROR2 immunoblot of HEK293 cells and HEK293 cells transfected with ROR2. A specific band of the right molecular weight was detected only on HEK-293 cells transfected with recombinant ROR2. B, anti-phosphotyrosine antibody control. HEK293 cells were transfected with ROR2 and stimulated for 15 min with 250 ng/ml Wnt5a. Cells were lysed in the absence or in the presence of a phosphatase inhibitor cocktail. ROR2 was immunoprecipitated from the cell lysate with anti-ROR2 antibody. The immunoprecipitate was then immunoblotted using a Phospho-Tyr–specific antibody. After phosphotyrosine detection, the membrane was stripped and reblotted with anti-ROR2 antibody to verify the target protein phosphorylation. C, ROR2 immunoprecipitation control. HEK293 cells expressing ROR2 were lysed and immunoprecipitated either with a control IgG or anti-ROR2 antibody. The immunoprecipitated was separated by SDS-PAGE and immunoblotted with a ROR2 antibody. [file mmc1.pdf]
